# *N*-Acyl Dopamines Induce Apoptosis in Endometrial Stromal Cells from Patients with Endometriosis

**DOI:** 10.3390/ijms221910648

**Published:** 2021-09-30

**Authors:** Alina M. Gamisonia, Marina N. Yushina, Irina A. Fedorova-Gogolina, Mikhail G. Akimov, Chupalav M. Eldarov, Stanislav V. Pavlovich, Vladimir V. Bezuglov, Natalia M. Gretskaya, Gennady T. Sukhikh, Mikhail Yu. Bobrov

**Affiliations:** 1 National Medical Research Center for Obstetrics, Gynecology and Perinatology Named after Academician V. I. Kulakov of the Ministry of Healthcare of Russian Federation, Akademika Oparina Str. 4, 117513 Moscow, Russia; alyafonya@mail.ru (A.M.G.); gmarinanikolaevna@gmail.com (M.N.Y.); irina.genet@gmail.com (I.A.F.-G.); chupalav@gmail.com (C.M.E.); st.pavlovich@mail.ru (S.V.P.); m_bobrov@oparina4.ru (G.T.S.); 2 Shemyakin-Ovchinnikov Institute of Bioorganic Chemistry, Russian Academy of Sciences, Miklukho-Maklaya Str. 16/10, 117997 Moscow, Russia; akimovmike@gmail.com (M.G.A.); vvbez@ibch.ru (V.V.B.); natalia.gretskaya@gmail.com (N.M.G.)

**Keywords:** endometrial stromal cells, endometriosis, *N*-acyl dopamines, endocannabinoid system, CB1 receptor, reactive oxygen species, selective toxicity, apoptosis

## Abstract

Endometriosis is characterized by the formation and development of endometrial tissues outside the uterus, based on an imbalance between proliferation and cell death, leading to the uncontrolled growth of ectopic foci. The potential target for the regulation of these processes is the endocannabinoid system, which was found to be involved in the migration, proliferation, and survival of tumor cells. In this paper, we investigated the effect of endocannabinoid-like compounds from the *N*-acyl dopamine (NADA) family on the viability of stromal cells from ectopic and eutopic endometrium of patients with ovarian endometriosis. *N*-arachidonoyldopamine, *N*-docosahexaenoyldopamine, and *N*-oleoyldopamine have been shown to have a five-times-more-selective cytotoxic effect on endometrioid stromal cells. To study the mechanisms of the toxic effect, inhibitory analysis, measurements of caspase-3/9 activity, reactive oxygen species, and the mitochondrial membrane potential were performed. It was found that NADA induced apoptosis via an intrinsic pathway through the CB1 receptor and downstream serine palmitoyltransferase, NO synthase activation, increased ROS production, and mitochondrial dysfunction. The higher selectivity of NADA for endometriotic stromal cells and the current lack of effective drug treatment can be considered positive factors for further research of these compounds as possible therapeutic agents against endometriosis.

## 1. Introduction

Endometriosis is a proliferative disease characterized by the formation of endometrial tissue outside of the uterus and its dissemination in the peritoneal cavity [1]. Endometriosis loci formation causes functional and structural changes in the affected organs, leading to chronic pain, infertility, and significant reduction in the quality of life [2].

The medical treatment of endometriosis exploits the endometrial tissue sensitivity to sex hormones and is thus based on the therapy that blocks ovarian function. The effect of such treatment is transient and often not sufficient to stop the disease. The alternative surgical resection of the visible lesions may be highly efficient but has a high incidence of recurrences [1]. In this regard, the search for an alternative noninvasive medical treatment that specifically targets the ectopic endometrial tissue is in great demand.

One of the potential targets for medical intervention is the endocannabinoid system (ECS), which has attracted attention over the last decade regarding its role in tumor cell growth and survival [3,4]. ECS components are present in the endometrium with different distribution between tissue compartments dependent on phases of the menstrual cycle [5]. In normal endometrium, ECS may have a regulatory action on endometrial decidualization and placentation [6]. It was also shown that the expression of ECS receptors and metabolic enzymes might change in endometriotic loci, indicating the possible role of endocannabinoids in endometriosis development [7]. 

The endocannabinoid system comprises endogenous cannabinoids, their receptors, and the enzymes engaged in their biosynthesis, transport, and degradation [8,9,10]. Endocannabinoids are derivatives of long-chain fatty acids, namely their amides with ethanolamine or ethers with glycerol. Among them, *N*-arachidonoylethanolamine (anandamide) and 2-arachdonoylglycerol (2-AG) were first discovered in the mammalian brain, and their physiological properties as neurotransmitters were thoroughly investigated [11]. Fatty acid amides with several amino acids, dopamine, and serotonin were subsequently characterized as endogenous compounds, but their biological role has been less studied so far [12,13,14]. The endocannabinoid effects may be mediated primarily through G-protein coupled cannabinoid receptors (CB1, CB2) and transient receptor potential vanilloid receptors (TRPV1), although other receptors have been recently described [15].

It was shown in numerous studies that anandamide, 2-AG, and their receptors might regulate the proliferation and migration of cancer cells from different origins [15]. Much less research has been conducted to elucidate the role of ECS in endometriosis. In an early work, Leconte et al. demonstrated that CB1 and CB2 receptors were expressed in cultured stromal cells derived from deep infiltrative endometriotic loci and that dual CB1/CB2 synthetic agonist WIN 55,212-2 mediated pronounced antiproliferative effect [16]. Another study showed that the same synthetic cannabinoid might elicit the cytotoxic effect in endometrial stromal cell lines [17]. There are few studies evaluating the direct effects of endocannabinoids on cells from endometriosis lesions. The toxic effect on endometriotic stromal cell viability was recently shown for endocannabinoid-related lipid *N*-arachidonoyl dopamine, but the cell death mechanisms were not investigated [18].

*N*-arachidonoyl dopamine is a member of the *N*-acyl dopamines (NADA) family, which comprises amides of long-chain fatty acids with dopamine found in mammalian brain tissues and human plasma [19]. Endogenous NADA are dopamine amides with arachidonic, oleic, stearic, and docosahexaenoic acids. NADA were characterized as ligands of cannabinoid and vanilloid receptors sharing pharmacological properties with other endocannabinoids [14,20]. Compared to anandamide and 2-AG, the significance of NADA under physiological and pathological conditions is not fully understood yet.

NADA were shown to be cytotoxic to different cancer cell lines representing major histological cancer types [21], thus indicating that these compounds may function as regulators of cell fate in case of an imbalanced proliferative phenotype. The NADA anticancer activity was also proved in a high-throughput screening study that enabled the identification of chemical compounds effective against glioblastoma multiforme stem cells [22]. A chemical library of 31,624 small molecules was tested, and 11 compounds were selected, among which *N*-arachidonoyldopamine and *N*-oleoyldopamine were presented.

In this paper, we evaluated the effects of the three most active natural *N*-acyl dopamines: *N*-arachidonoyldopamine (AA-DA), *N*-docosahexaenoyldopamine (DHA-DA), and *N*-oleoyldopamine (OL-DA) on the viability of primary stromal cells derived from the ectopic and eutopic endometrium of patients with ovarian endometriosis. NADA demonstrated selective toxicity for ectopic stromal cells. The substances induced apoptosis via the intrinsic pathway with the involvement of CB1 receptor, serine palmitoyltransferase, NO-synthase, ROS production, and mitochondrial dysfunction.

## 2. Results

### 2.1. Effect of N-Acyl Dopamines on the Viability of Endometriotic Stromal Cells

**Figure 1:**
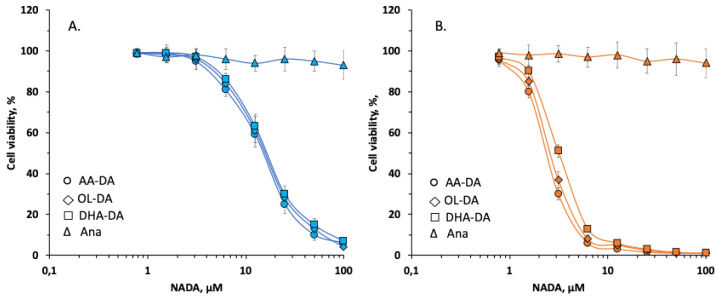
Dose-dependent cytotoxic effect of *N*-acyl dopamines on stromal cells of eutopic (**A**) and ectopic (**B**) endometrium. Cell viability was measured using MTT-test as described in Section 4. Data are presented in percent from vehicle-treated controls as mean ± standard deviation.

**Figure 2:**
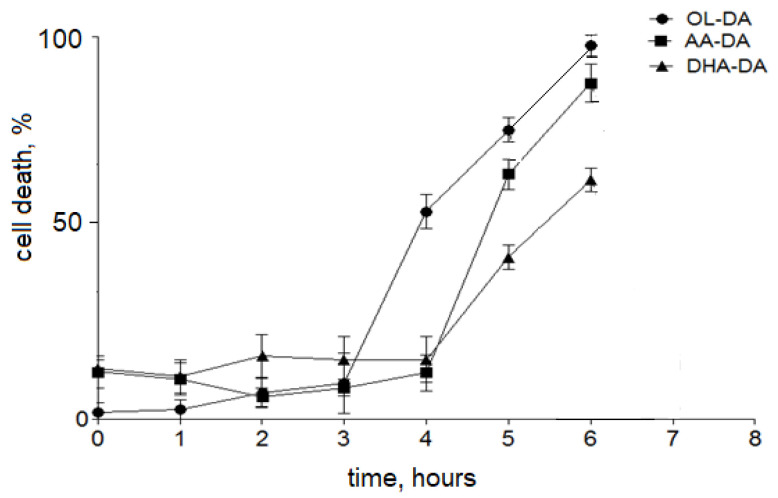
Kinetics of cytotoxic action of *N*-acyl dopamines on stromal cells of ectopic endometrium. Cell viability was measured after indicated time intervals (NADA 10 μM) as described in Section 4. Data are presented as mean ± standard deviation.

The effects of dopamine amides of arachidonic (AA-DA), docosahexaenoic (DHA-DA), and oleic (OL-DA) acids on the cell viability were tested in cultures of stromal cells derived from eutopic endometrium and endometriotic cysts of patients with ovarian endometriosis. After 24 h incubation NADAs demonstrated a moderate cytotoxic effect on stromal cells of the eutopic endometrium (EuSC) in the concentration range of 0.78–100 μM. All compounds were equally potent with a half-lethal concentration (LC_50_) ranging in 14.06–16.02 μM (Figure 1A, Table 1). Stromal cells of the ectopic endometrium (EcSC) showed higher sensitivity to the NADAs cytotoxicity, with the considerable decrease of LC_50_ values down to 2.45–3.24 μM range (Figure 1B, Table 1). The data indicate that the structure of NADAs fatty acid moiety does not drastically influence their effects on cell viability in the used conditions. However, AA-DA and OL-DA showed some higher selectivity compared to DHA-DA. Endogenous cannabinoid anandamide (Ana) was not cytotoxic to EuSC and EcSC at the concentrations tested (Figure 1A,B). The kinetics of the cytotoxic effect of active compounds was also investigated on stromal cells from ectopic endometrium. OL-DA was the most potent with the onset of cytotoxic action after 3 h and maximal effect after 6 h of incubation (Figure 2). The toxic effects of the other two NADAs were visible after the fourth hour of incubation, but AA-DA had faster kinetics compared to DHA-DA (Figure 2). It was shown that all NADAs had a pronounced effect on cell viability after 5 h of incubation; thus, this time interval was chosen for further studies of the mechanisms of NADA cytotoxic action.

### 2.2. The Mechanism of Cytotoxic Effect of N-Acyl Dopamines

To identify receptors responsible for NADAs cytotoxicity, stromal cells of ectopic endometrium were preincubated with antagonists of CB1 (SR141716A), CB2 (SR144528), and TRPV1 (capsazepine) receptors. The blockade of the CB1 receptor considerably increased cell viability in the presence of NADA, while CB2 and TRPV1 antagonists had no effect (Table 2). To evaluate the intercellular mechanisms of NADA toxicity, we used several modulators of possible molecular targets involved in cell death induced by cannabinoids [3]: pan-caspase inhibitor Z-VAD-FMK, serine palmitoyltransferase inhibitor (SPT) imideazopyridine, NO-synthase (NOS) inhibitor L-NAME, and antioxidants *N*-acetyl-L-cysteine and ascorbic acid. It was shown that pan-caspase and SPT inhibitors prevented NADA cytotoxicity (Table 2), indicating the involvement of apoptotic cascades associated with ceramide formation and caspases activation. To the same extent, the use of *N*-acetyl-L-cysteine and ascorbic acid was able to protect stromal cells in toxic conditions that may be the evidence of the reactive oxygen species production under the NADA treatment. The effect of L-NAME was less pronounced, but the inhibition of the NO-synthase markedly increased cell viability in the presence of NADA. Several tests were performed further to confirm the apoptotic cell death and ROS production.

### 2.3. Annexin-Propidium Iodide Assay

To determine the type of NADA-induced cell death, stromal cells of the ectopic endometrium were stained with fluorescently labeled annexin and propidium iodide (PI). The fluorescent dye binding analysis and cell counting were performed using flow cytometry. The increase in annexin fluorescence indicates the rise of phosphatidylserine content in the outer surface of the plasma membrane that is a characteristic feature of apoptotic cell death. In turn, propidium iodide fluorescence evidences the disruption of the plasma membrane integrity specific to necrotic cell death.

**Figure 3:**
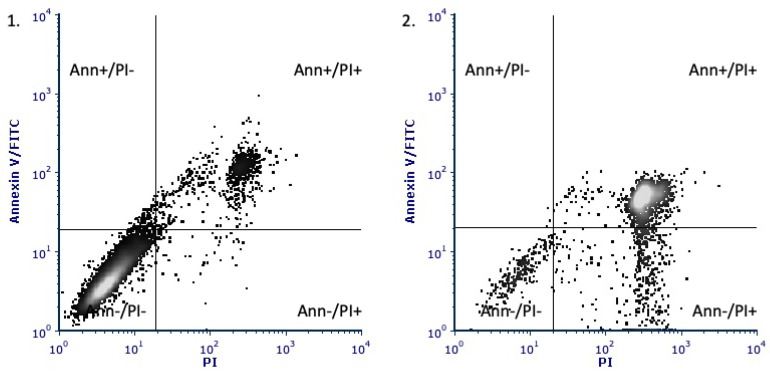
The evaluation of the type of NADA-induced ectopic stromal cell death with annexin V-FITC/PI assay. A. Representative plots of fluorescence distribution in ectopic stromal cells incubated with DMSO (**1**) or AA-DA (**2**).

After the NADA treatment, a signal from labeled annexin alone (Annexin V+/PI−) indicating an early stage of apoptosis was recorded in a small number of cultured cells. A combination of signals from both labels (Annexin V+/PI+, the late apoptosis) and PI alone (Annexin V−/PI+, necrosis) was registered in 62–68% and 18–22% of cells correspondingly (Figure 3, Table 3). The data may indicate that more than fifty percent of stromal cells undergo apoptosis after NADA treatment.

### 2.4. Caspase Activity Assay

To confirm the caspase-dependent pathway of cell death, the activities of caspase-3 and -9 were measured using specific fluorescent substrates in the preparations from stromal cells exposed to NADA treatment. After the NADA treatment, a marked increase in the fluorescence intensity in cell lysates was observed compared to the nontreated control, indicating the caspase activation. The use of pan-caspase inhibitor Z-VAD-FMK completely inhibited NADA-induced caspase activity (Table 4). Thus, caspase-3 and -9 are the effectors of NADA induced apoptosis.

### 2.5. Effect of N-Acyl Dopamines on ROS Production and Mitochondrial Membrane Potential

**Figure 4:**
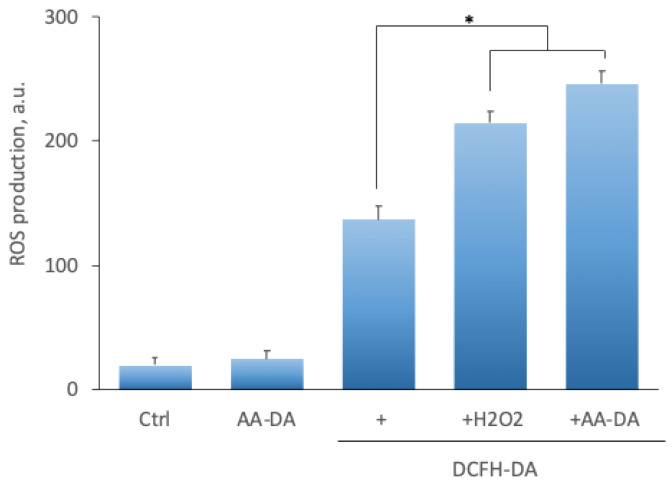
Representative plot of AA-DA effect on ROS production by endometriotic stromal cells. ROS production was measured using a 2,7-dichlorodihydrofluorescein diacetate (DCFH-DA) in the presence of AA-DA (5 μM) or H_2_O_2_ (5 μM). *—statistically significant difference, *p* < 0.05, ANOVA with the Tukey post-hoc test.

**Figure 5:**
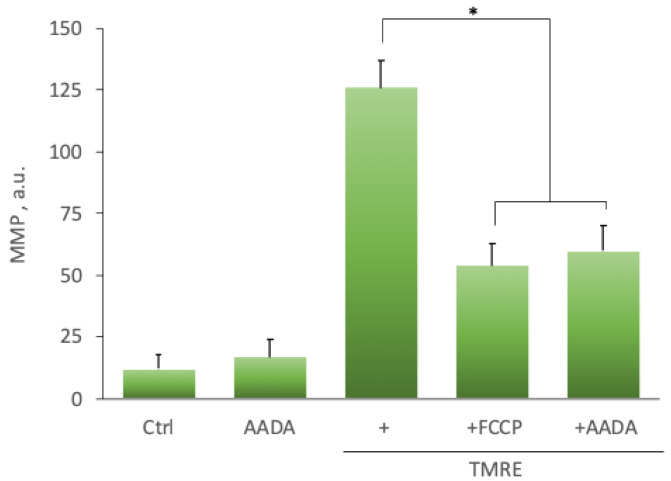
Representative plot of AA-DA effect on the mitochondrial membrane potential in ectopic stromal cells. The mitochondrial membrane potential (MMP) was assessed by a fluorometric assay using tetramethylrhodamine ethyl ester (TMRE) in the presence of AADA 5 μM or FCCP 20 μM. *—a statistically significant difference, *p* < 0.05, ANOVA with the Tukey post-hoc test.

Our data on the blockade of NADA toxicity by antioxidants suggest that one of the cell death mechanisms may be NADA-induced generation of reactive oxygen species (ROS). ROS production was measured using a 2′,7′-dichlorodihydrofluorescein diacetate (DCFH-DA) fluorescent indicator. DCFH-DA is a fluorogenic dye to measure hydroxyl, peroxyl, and other ROS within the cell. Esterases deacetylate DCFH-DA, and ROS further oxidizes the resulting compound into highly fluorescent 2′,7′-dichlorofluorescein (DCF). In the ectopic stromal cell cultures, NADA addition (5 µM) significantly increased dye fluorescence, and this effect was comparable with those observed after the addition of 5 µM H_2_O_2_ (Figure 4, Table 5). One of the reasons for ROS generation may be the impairment of mitochondrial function. We tested this possibility using tetramethylrhodamine ethyl ether, a cationic dye readily sequestered by active mitochondria. In the presence of NADA, a significant decrease in TMRE fluorescence was observed; the use of the mitochondrial membrane protonophore FCCP, which uncouples oxidative phosphorylation, reduced TMRE signal (Figure 5, Table 6). These findings suggest that NADA may affect mitochondrial activity, at least in part, by altering the mitochondrial membrane potential, leading to ROS production and the development of an apoptotic cascade.

## 3. Discussion

Previously, it was hypothesized that endometrial cells implant and develop lesions due to cell adhesion and mobility changes and disturbances in the regulation of proliferation and apoptosis [23]. Although described in many other tissue models, the direct effects of endocannabinoids and related endogenous lipids on endometriotic cells remain mostly unexplored. To address this issue, we used primary cultures of stromal cells from ovarian endometrioma and eutopic endometrium to investigate the effects of *N*-acyldopamines on endometrial stromal cell viability. In our experimental setting, NADA dose-dependently induced stromal cell death with higher specificity to cells from ectopic endometrium (Figure 1). We found a five-fold increase in sensitivity of endometriotic stromal cells to all NADA tested after 24 h treatment. The onset of submaximal toxicity was observed after 5 to 6 h of incubation, and NADA showed different kinetics of toxic effect with the following rank of activity: OL-DA > AA-DA > DHA-DA (Figure 2). The cytotoxicity was mediated through CB1 receptors as it was blocked by the application of the CB1 antagonist, while CB2 and TRPV1 antagonists were inactive (Table 2).

The expression of CB1 and CB2 receptors has been reported in the glandular epithelium and the stromal cells of normal endometrium [5]. TRPV1 receptor was also found on endometrial stromal cells and together with CB1 on nerve fibers [24,25]. In the eutopic and ectopic endometrium of women with endometriosis and adenomyosis, lower expression of CB1 and CB2 was shown compared to normal tissues [26,27]. In contrast, TRPV1 expression was shown to increase in ovarian endometrioma, and stimulation of stromal cells by proinflammatory factors may induce TRPV1 expression [24]. This data indicate that receptors of the endocannabinoid system may have functional significance in normal and pathological endometrium.

In cultured stromal cells derived from ectopic, eutopic, and normal endometrium, no significant difference in CB1/CB2 expression was found [16]. In this single study, CB1/CB2 mediated effects on proliferation and viability of stromal cells from deep infiltrating endometriotic loci were investigated. The direct action of CB1/CB2 agonist WIN 55,212-2 dose-dependently decreased proliferation of stromal cells from ectopic, eutopic, and normal endometrium but did not affect cell viability in concentrations up to 40 μM. In this study, cannabinoid receptor antagonists were not used to determine the type of CB receptor involved in the effect. Antiproliferative and pro-apoptotic effects of the CB1/CB2 receptors activation were also investigated on two cell lines from endometrial adenocarcinoma (Ishikawa cell line) and ovarian endometrioma (CRL-7566 immortalized stromal cells). Synthetic agonist of CB1 exhibited a stronger antiproliferative effect on Ishikawa cells and pro-apoptotic effects on CRL-7566 cells. The CB2 agonist caused stronger anti-proliferation on CRL-7566 cells and pro-apoptotic effects on Ishikawa cells [28]. The properties of endocannabinoid anandamide were investigated using the St-T1b cell line (immortalized stromal cells from endometrium). In this study, anandamide was cytotoxic, but the effect was not modulated by CB1, CB2, or TRPV1 antagonists [29]. Interestingly, in the same St-T1b cell line, synthetic CB1/CB2 agonist WIN 55,212-2 induced proliferation blockade and caused toxicity through the CB1 receptor [17].

In this work, endocannabinoid anandamide was nontoxic in stromal cell cultures from both ectopic and eutopic endometrium (Figure 1). It is difficult to explain why CB1 agonists anandamide in our study and WIN 55,212-2 in previous work [16] were inactive, while NADA promoted cytotoxicity at least in part through CB1 activation. However, such differences in cannabinoid receptor ligands activity have already been described, although without a mechanistic explanation. In a recent study, Fonseca et al. showed that among three potent synthetic CB1 agonists JWH-122, UR-144, and WIN55,212-2, only the latter induced mitochondrial dysfunction and apoptosis in endometrial stromal cells, while others stimulated prompt ROS formation and endoplasmic reticulum stress without reduction in cell viability [17].

In a recent study, the antitumor activity of several NADA, anandamide, and WIN 55212-2 was tested in a RAS-activated model of onco-transformation on Ba/F3 cell line [30]. Again, NADA potently decreased proliferation and cell viability in RAS-transformed cells, while anandamide and WIN 55,212-2 were inactive. It was shown that NADA activity was associated with the inhibition of membrane translocation of RAS family member NRAS, and this effect was not observed in the presence of anandamide. Authors speculated that observed NADA inhibition of viability and proliferation was independent of CB-receptors activation. This data indicates that NADA may have additional mechanisms beyond the ECS mediated activity and that interaction with CB1 alone may not be sufficient to induce intracellular responses leading to activation of death programs.

The investigation of intracellular events associated with NADA toxicity to endometrioid cyst stromal cells revealed the possible involvement of the intrinsic apoptosis pathway. This conclusion was supported by the anticytotoxic effects of serine palmitoyltransferase (SPT) and pan-caspase inhibitors (Table 2), as well as by the increase in the number of annexin positive cells (Figure 3, Table 3) and activation of caspases-9 and its effector caspase-3 (Table 4). The SPT activity is responsible for de novo ceramide synthesis and regulation of signaling cascades controlling the growth and survival of tumor cells [31,32]. The ability of CB1 agonists to stimulate SPT was shown in glioma cell lines. Treatment with plant cannabinoid delta-(9)-Tetrahydrocannabinol induced apoptosis and ceramide production, which was prevented by SPT inhibitor [33]. The induction of apoptosis by endocannabinoids through the intrinsic pathway activation has also been shown in several previous studies. It has been reported that in rat decidual cells, anandamide induced apoptosis through CB1-dependent increase in ceramide production, caspases (-9, -3/7) activation, and p38 mitogen-activated protein kinase (MAPK) phosphorylation [34]. Anandamide-induced cytochrome C release and caspases activation were shown in rat pheochromocytoma PC12 cells. These effects resulted from activating p38 MAPK and c-Jun *N*-terminal kinase (JNK) pathways [35]. In glioma cell lines, anandamide treatment led to cytochrome C release and apoptosis via CB1, dependently on lipid raft integrity [36].

In our study, NADA cytotoxicity was also inhibited by two antioxidants, *N*-acetylcysteine and ascorbate (Table 2), indicating a possible involvement of reactive oxygen species production and oxidative stress in NADA-induced cell death. We found a significant increase in ROS generation in the presence of NADA comparable with the application of 5 µM of H_2_O_2_ (Figure 4, Table 5). ROS production may be the consequence of mitochondrial dysfunction, a typical intrinsic apoptosis induction pathway component. NADA induced a significant decrease in mitochondrial function, which was confirmed by measuring mitochondrial membrane potential (Figure 5, Table 6).

Previously, it has been shown that cannabinoids can stimulate mitochondrial ROS generation in different cells, leading to the impairment of mitochondrial function and cytochrome C release [37,38,39]. In rat decidual cells, CB1 activation led to ROS generation related to increased ceramide synthesis [39]. In the BeWo cell line (originated from human trophoblast cells), synthetic cannabinoids induced ROS production, mitochondrial dysfunction, and caspase-9 activation [40]. The direct effects of cannabinoids were shown in rat heart mitochondria, where they caused significant decreases in oxygen consumption and mitochondrial membrane potential and an increase in mitochondrial hydrogen peroxide production. In the case of NADA, the alternative action cannot be excluded. Owing to their lipophilic nature, NADA may enter the cell and interact with intracellular targets such as mitochondria. It was described previously that NADA dopamine moiety might donate protons with the possible formation of quinone isoforms [41]. It was also shown that dopamine oxidation leads to the production of quinone metabolites, such as aminochrome, which can form adducts with mitochondrial proteins [42], inducing mitochondrial dysfunction and a subsequent collapse in energy. Incorporating to mitochondrial membrane NADA may act as uncouplers that in turn may lead to depolarization and ROS production, although this proposal needs to be confirmed. In a recent study, it was shown that NADAs were toxic to several cancer cell lines, and this effect was at least in part mediated through NO-synthase activation and NO production [43]. In our study, we have also observed the possible involvement of this mechanism as the use of NO-synthase inhibitor (L-NAME) increased stromal cell viability. However, this effect was not as pronounced as for ascorbate and *N*-acetylcysteine.

In this study, we found a higher selectivity of the cytotoxic action of *N*-acyl dopamines against ectopic endometrial stromal cells from ovarian cysts. However, the data obtained do not allow us to state unequivocally that a similar selectivity can be observed for endometrial epithelial cells or stromal cells from endometrioid foci of other localizations. We are also aware that the activity of *N*-acyl dopamines shown here in vitro may not be reproduced in vivo due to the unexplored parameters of the pharmacodynamics and pharmacokinetics of these compounds. Further research is needed to remove the limitations of this work.

Based on the presented results, it may be concluded that in stromal cells from ectopic endometrium of ovarian cysts, NADA cytotoxicity is characterized by a typical CB1-mediated apoptosis pathway [44], including downstream ceramide and ROS production leading to caspase-9/-3 activation and mitochondrial dysfunction. Taken together with the previous findings on the NADA pronounced analgesic and anti-inflammatory effects [19,45] and our data on the higher NADAs specificity to stromal cells from endometriotic ovarian cysts, these properties may be considered as a positive factor for further research of these compounds as possible therapeutic agents for endometriosis treatment.

## 4. Materials and Methods

### 4.1. Sample Collection

Biopsies of eutopic endometrium and endometriotic cysts were obtained from patients undergoing surgical treatment for ovarian endometriosis. Ethics approval for the study was obtained from the ethics committee at the National Medical Research Center for Obstetrics, Gynecology, and Perinatology, named after Academician V.I.Kulakov (protocol №9 from 16.11.2017, Ministry of Healthcare, Russian Federation). Written informed consent was obtained from each patient under study. 

Tissues were collected under sterile conditions and transferred to the laboratory in a DMEM/F-12 (1:1) incubation medium without serum containing 2 mM glutamine, 100 U/mL penicillin, 100 μg/mL streptomycin, and 0.25 μg/mL amphotericin B (medium and additives were from PanEco, Russia). Cell isolation was performed not more than 30 min after tissue collection. The presence of endometriotic lesions in the portions of collected biopsies was confirmed by a pathologist after histological examination.

### 4.2. Cell Isolation and Culture

Primary endometrial stromal cell cultures were prepared from paired tissue samples as described previously [46]. Briefly, biopsy specimens were washed with phosphate-buffered saline to remove blood and debris and then minced into small pieces. Samples were placed into a CO_2_-incubator (5% CO_2_, 37 °C) and digested with collagenase IA (0.2%, Sigma-Aldrich, Burlington, MA, USA) in the incubation medium DMEM/F-12 (1:1) without serum, 2 h for ectopic or 30 min for eutopic endometrium. The debris and epithelial glands were removed by sequential filtration through 100 μm and 40 μm cell strainers (SPL Lifesciences, Pocheon-si, Korea). Cells were washed in an incubation medium with 10% fetal bovine serum, and further seeded onto cell culture flasks (Corning, Corning, NY, USA), and cultured in CO_2_-incubator (5% CO_2_, 37 °C). All experiments were performed on primary cell cultures at the third passage, and the various tests were performed in triplicates on stromal cell lines from at least three patients.

### 4.3. Cell Viability Assays

The cells that had reached confluence were detached with 0.25% trypsin in Hanks’ balanced salts solution with 0.53 mM EDTA (Trypsin-EDTA, PanEco, Moscow, Russia), washed, and resuspended in the serum-containing incubation medium. Then cells were seeded in 96-well plates (Corning) with a final density of 30 × 10^3^ cells per well and incubated for 24 h. Then NADAs in various concentrations (0.78–100 μM) or vehicle (DMSO) were added for viability assays, and cells were incubated for 24 h. The serial dilutions of the tested compounds were prepared in DMSO and then dissolved in the cultivation medium so that DMSO concentration was 0.05%. The compounds were added to cells in triplicate for each concentration, and control cells were treated with 0.05% DMSO alone. *N*-acyl dopamines and anandamide (*N*-arachidonoyl-ethanolamine, AEA) were synthesized in the Laboratory of Oxylipins of Shemyakin-Ovchinnikov Institute of Bioorganic Chemistry (Moscow, Russia) as described previously [47] and stored under an argon atmosphere at −80 °C. Before the experiments, a purity check of test compounds was performed using reversed-phase HPLC, and only stock solutions with purity not less than 98% were used.

Cell viability and kinetics of cytotoxic effect were analyzed by MTT-assay based on the MTT dye (4,5-dimethylthiazol-2-yl)-2,5-diphenyltetrazolium bromide reduction by mitochondria of living cells to colored formazan. For the assay, the medium with the test compounds was removed, and cells were incubated for 1 h in Hanks’ balanced salts solution, supplemented with 10 mM of D-glucose and 0.5 mg/mL of MTT (PanEco). After the incubation, the solution was removed, and cells were dissolved in DMSO. The amount of the reduced dye was determined at 594 nm with a reference wavelength of 620 nm using an EFOS 9505 photometer (Sapphire, Moscow, Russia).

### 4.4. Inhibitory Analysis of N-Acyl Dopamines Potential Targets and Effectors

Ectopic stromal cells were seeded in 96-well plates (30 × 10^3^ per well) and incubated as described above with *N*-acyl dopamines in combination with modulators of their possible effector targets: antagonists of cannabinoid receptor type 1 SR141716A (0.5 μM) and type 2 SR144528 (0.5 μM), an antagonist of TRPV1 receptor capsazepine (2 μM), antioxidants *N*-acetyl-L-cysteine (5 μM) and ascorbic acid (50 μM), NO-synthase inhibitor L-NAME, imideazopyridine (5 μM) serine palmitoyltransferase (SPT) inhibitor (all compounds listed were from Sigma-Aldrich, USA) and 50 μM pan-caspase inhibitor Z-VAD-Fmk (*N*-Benzyloxycarbonyl-Val-Ala-Asp(O-Me) fluoromethyl ketone); Tocris Bioscience, Bristol, UK). Tested compounds were added 1 h prior to *N*-acyl dopamines. After the incubation, cell viability was analyzed by MTT-assay.

### 4.5. Cell Death Annexin V-FITC/PI Assay 

For the assay, cells (10^5^ per well) were seeded in 24-well plates (Corning), incubated for 24 h, and *N*-acyl dopamines (5 μM) were added for the next 5 h. Then stromal cells were harvested with trypsin-EDTA solution, washed twice with cold PBS (800 g for 5 min), and the pellet was resuspended in binding buffer (10 mM HEPES/NaOH pH 7.4, 140 mM NaCl, 2.5 mM CaCl_2_). Cell suspension (100 μL) was transferred to a 5 mL culture tube and incubated with 5 μL of FITC-conjugated annexin V (1 mg/mL, Sigma-Aldrich, USA) and 5 μL of propidium iodide (PI, 2 mg/mL, Sigma-Aldrich) for 15 min at room temperature in the dark. A total of 400 μL of the binding buffer was added to each sample tube, and cell counts were performed by FACSCalibur flow cytometer (BD Biosciences, Franklin Lakes, NJ, USA). The distribution of fluorescent dyes was analyzed using Cell Quest Research Software (BD, Biosciences). Cells were classified as live (Annexin V−, PI−), necrotic (Annexin V−, PI+), early apoptotic (Annexin V+, PI−), and late apoptotic (Annexin V+, PI+). 

### 4.6. Caspase-3 Activity and Caspase-9 Activity Measurement

Caspase activity was determined in cell lysates by fluorometric measurement of 7-amino-4-trifluoromethyl coumarin (AFC) release from the fluorogenic caspase-3 substrate Ac-DEVD-AFC (Ac-Asp-Glu-Val-Asp-AFC, Sigma-Aldrich) and caspase-9 substrate Ac-LEHD-AFC (Ac-Leu-Glu-His-Asp-AFC, Sigma-Aldrich). After 5-h incubation in the presence or absence of *N*-acyl dopamines, cells (10^5^ per well) incubated in 24-well plates were washed in PBS and lysed in 50 μL lysis buffer (10 mM HEPES, pH 7.4, 2 mM EDTA, 0.1% CHAPS, 5 mM DTT, supplemented with 10 μg/mL pepstatin, 10 μg/mL aprotinin, 20 μg/mL leupeptin, and 1 mM PMSF (Sigma-Aldrich)). Further lysates were centrifuged 30 min at 13,000× *g* at room temperature and diluted 500-fold in the reaction buffer 10 mM HEPES, pH 7.4, 2 mM EDTA, 0.1% CHAPS, and 5 mM DTT. Sample aliquots (190 μL) in triplicates were transferred to 96-well plates, and 10 μL of substrate solution (0.2 mg/mL) was added to each well. The fluorescence intensity (excitation wavelength, 390 nm; emission at 520 nm) of the samples was measured in 1-min intervals immediately after the substrate addition using Hidex Sense Beta Plus microplate reader (Hidex, Turku, Finland). A pan-caspase inhibitor Z-VAD-FMK (50 μM) was used as a negative control. Samples were obtained from three stromal cell lines, in triplicates for each tested compound.

### 4.7. DCFH-DA Reactive Oxygen Species ROS Assay 

Cells (10^5^ per well) were incubated in 24-well plates as described in the previous section. After 5 h of incubation with *N*-acyl dopamines, cells were detached with trypsin-EDTA solution, washed with PBS, and incubated for 30 min with 10 μM of 2′-7′-dichlorofluorescein diacetate (DCFH-DA, Sigma-Aldrich, USA) at room temperature. Control cell cultures were incubated without NADAs or in the presence of hydrogen peroxide (5 μM). 2′,7′-dichlorofluorescein (DCF) fluorescence was assayed at 530 nm after excitation of cells at 488 nm using FACSCalibur flow cytometer (BD Biosciences).

### 4.8. Measuring Mitochondrial Transmembrane Potential by TMRE Staining

The mitochondrial membrane potential was assessed by a fluorometric assay using tetramethylrhodamine ethyl ester (TMRE). Stromal cells were treated with *N*-acyl dopamines as described above and then exposed to TMRE (500 nM, Sigma-Aldrich) for 30 min at room temperature. A total of 20 μM of FCCP (cyanide trifluoromethoxyphenlhydrazone, Sigma-Aldrich) was added before TMRE addition as a positive control. Changes in dye fluorescence were analyzed by FACSCalibur flow cytometer (BD Biosciences). 

### 4.9. Statistical Analysis 

Data analysis was performed using GraphPad Prism and Microsoft Excel software. Data are presented as mean ± standard deviation. Data were compared using the unpaired Student’s *t*-test for pairwise comparison and ANOVA with the Tukey post-test for multiple comparisons; *p* values of 0.05 or less were considered significant. 

## Figures and Tables

**Table 1:** LC_50_ values of NADA cytotoxic effect on eutopic (EuSC) and ectopic (EcSC) stromal cells.

Compounds	EuSC (LC_50_, µM)	EcSC (LC_50_, µM)
OL-DA	15.03 ± 0.97	2.72 ± 0.55
AA-DA	14.06 ± 0.98	2.45 ± 0.45
DHA-DA	16.02 ± 0.99	3.24 ± 0.50
Ana	-	-

Data are presented as mean ± standard deviation.

**Table 2:** Inhibitory analysis of potential effectors of NADA toxicity.

Compounds		OL-DA	AA-DA	DHA-DA
NADA		21.4 ± 2.9	22.9 ± 1.9	29.1 ± 2.3
NADA + SR141716A	CB1	70.2 ± 3.4 *	73.2 ± 3.0 *	75.1 ± 1.4 *
NADA + SR144528	CB2	19.4 ± 2.4	18.2 ± 1.4	23.3 ± 3.4
NADA + Capsazepine	TRPV1	24.1 ± 1.3	27.1 ± 1.6	27.9 ± 2.9
NADA + Z-VAD-FMK	Casp	86.2 ± 3.9 *	81.8 ± 1.9 *	81.2 ± 1.3 *
NADA + *N*-acetyl-L-cysteine	ROS	72.2 ± 2.6 *	70.3 ± 2.9 *	74.4 ± 1.4 *
NADA + Ascorbate	ROS	74.8 ± 2.7 *	76.4 ± 2.1 *	79.3 ± 2.6 *
NADA + Imideazopyridine	SPT	78.4 ± 2.3 *	78.6 ± 2.7 *	74.2 ± 2.4 *
NADA + L-NAME	NOS	42.1 ± 1.9	36.2 ± 4.7	39.4 ± 2.1

Ectopic stromal cells were incubated with NADA (5 μM) and blockers as described in Section 4. Cell viability was measured using MTT-test after 24 h incubation. Values of cell viability are presented as a percent from intact control. Data are presented as mean ± standard deviation; *—a statistically significant difference from NADA without blockers *p* < 0.05, ANOVA with the Tukey post-test. NADA—*N*-acyl dopamines, SR 141716A (CB1 antagonist) 0.5 μM, SR 144528 (CB2 antagonist) 0.5 μM, capsazepine (TRPV1 antagonist) 2 μM, Z-VAD-FMK (pan-caspase inhibitor) 50 μM, *N*-acetyl-L-cysteine 5 μM and ascorbic acid 50 μM (antioxidants), L-NAME (NO-synthase inhibitor), imideazopyridine 5 μM (SPT inhibitor).

**Table 3:** The ratio of labeled cells after treatment with NADA.

Cell Populations	OL-DA	AA-DA	DHA-DA	Control
Annexin V−/PI−, vital cells	12.5 ± 3.1	10.6 ± 2.7	16.2 ± 2.2	88.4 ± 2.6
Annexin V+/PI−, early apoptosis	2.1 ± 0.4	1.4 ± 0.6	1.2 ± 0.4	1.4 ± 0.7
Annexin V+/PI+, late apoptosis	68.2 ± 6.4	66.5 ± 4.2	62.3 ± 4.8	9.6 ± 2.5
Annexin V−/PI+, necrosis	18.4 ± 3.2	22.8 ± 2.4	21.6 ± 2.1	1.3 ± 0.4

Data presented as the percent of differently labeled cell populations (mean ± standard deviation). Annexin V/PI: Ann−/PI− vital population, Ann+/PI−: early apoptosis population, Ann+/PI+: late apoptosis population, Ann V−/PI+: necrosis population, damaged cells. Ectopic stromal cells were incubated for 5 h with NADA (5 μM), labeled, and counted with flow cytometry.

**Table 4:** The effect of NADA treatment on caspase-3 and -9 activity in ectopic stromal cells.

	OL-DA		AA-DA		DHA-DA	
	casp-3	casp-9	casp-3	casp-9	casp-3	casp-9
Control	134.2 ± 6.4	142.4 ± 4.2	121.3 ± 5.1	128.7 ± 5.1	137.6 ± 7.2	144.3 ± 3.9
Z-VAD-FMK	141.6 ± 6.3	142.1 ± 3.8	134.2 ± 3.3	132.2 ± 4.9	147.5 ± 5.3	144.8 ± 4.4
NADA	434.1 ± 5.9 *	450.8 ± 6.9 *	432.4 ± 4.2 *	465.2 ± 8.4 *	439.2 ± 7.9 *	470.8 ± 5.5 *
NADA+Z-VAD-FMK	137.4 ± 5.2	145.9 ± 4.3	129.1 ± 3.2	139.4 ± 5.8	138.8 ± 6.2	143.2 ± 6.4

Caspase activity in the vehicle (Control) or NADA (10 μM) treated stromal cells in the absence or presence of pan-caspase inhibitor Z-VAD-FMK (50 μM). Caspase activity was measured in cell preparations in the presence of fluorogenic substrates Ac-DEVD-AFC for caspase-3 and Ac-LEHD-AFC for caspase-9 as described in Materials and Methods. Data are presented in arbitrary units (a.u.) of fluorescence intensity as mean ± standard deviation. *, a statistically significant difference from Control, *p* < 0.05, Student’s *t*-test.

**Table 5:** Effects of NADA on the endogenous ROS production by ectopic stromal cells.

	OL-DA	AA-DA	DHA-DA
Control	19.3 ± 5.4	20.1 ± 5.9	21.9 ± 7.2
NADA	24.2 ± 7.8	25.4 ± 6.7	27.3 ± 5.3
DCFH-DA	134.3 ± 9.2	136.8 ± 10.8	138.5 ± 6.4
DCFH-DA + H2O2	220.2 ± 6.4 *	215.3 ± 9.4 *	221.4 ± 7.9 *
DCFH-DA + NADA	241.4 ± 8.9 *	246.1 ± 9.7 *	250.5 ± 7.1 *

Data are presented in arbitrary units (a.u.) of fluorescence intensity as mean ± standard deviation. *—a statistically significant difference from DCFH-DA treatment along, *p* < 0.05, ANOVA with the Tukey post-hoc test.

**Table 6:** The influence of NADA treatment on mitochondrial membrane potential in ectopic stromal cells.

	OL-DA	AA-DA	DHA-DA
Control	10.4 ± 4.4	12.1 ± 3.3	12.8 ± 6.2
NADA	15.2 ± 3.2	16.8 ± 3.2	18.3 ± 4.7
TMRE	125.3 ± 3.4	125.9 ± 5.8	128.2 ± 4.7
TMRE + FCCP	51.4 ± 5.2 *	54.3 ± 4.4 *	55.9 ± 4.3 *
TMRE + NADA	47.1 ± 4.3 *	59.8 ± 5.1 *	48.7 ± 3.4 *

Data are presented in arbitrary units (a.u.) of fluorescence intensities as mean ± standard deviation. *—a statistically significant difference from TMRE treatment along, *p* < 0.05, ANOVA with the Tukey post-hoc test.

## Data Availability

The data presented in this study are available on request from the corresponding author.
